# A simple and low-cost environmental enrichment program improves the welfare of *Calomys callosus*, a species that adapts to animal facilities

**DOI:** 10.3389/fvets.2024.1436907

**Published:** 2024-09-13

**Authors:** Sandra Gabriela Klein, Tamires Soares de Assis, Gabriel Silva Pereira, Loyane Bertagnolli Coutinho, Renan Faria Guerra, Matheus Morais Neves, Flávia Batista Ferreira, Isabela Lemos de Lima, Richard Costa Polveiro, Eloisa Amália Vieira Ferro, Murilo Vieira da Silva

**Affiliations:** ^1^Biotechnology in Experimental Models Laboratory - LABME, Universidade Federal de Uberlândia, Uberlândia, Brazil; ^2^Rodents Animal Facilities Complex, Universidade Federal de Uberlândia, Uberlandia, Brazil; ^3^Institute of Biomedical Sciences, Universidade Federal de Uberlândia, Uberlandia, Brazil

**Keywords:** *C. callosus* reproduction, animal welfare, environmental enrichment, precocity in females, experimental model

## Abstract

An environmental enrichment protocol is essential for testing experimental models because it upholds animal welfare, aligns with ethical principles in animal experimentation, and reduces the number of animals needed. *Calomys callosus*, a South American rodent from the Cricetidae family, is bred in rodent animal facilities for its ease of handling, longevity, prolificacy, and effectively mimicking diseases like Toxoplasmosis, Leishmaniasis, Chagas, and Schistosomiasis. There are no reports on environmental enrichments for this species or their impact on reproductive parameters. This study aimed to analyze the influence of the Environmental Enrichment Program (EEP) on the reproductive and zootechnical performance of *C. callosus* kept in the Rodents Animal Facilities Complex of Universidade Federal de Uberlândia (UFU). Two experimental groups were established: with environmental enrichment EE+ and without environmental enrichment EE−. The materials used in the experimental design were changed weekly and alternated between dietary, occupational, physical/cognitive, and non-enrichment items. After the inclusion of the EEP, an improvement in the reproductive indices of *C. callosus* was identified in the EE+ group. These improvements included increased female precocity, a decreased interbirth interval, and a higher number of pairs producing more offspring. The postpartum zootechnical indices were also better, such as the number of animals born alive, improved weaning rates, and a reduced average number of deaths from birth to weaning. After the inclusion of the EEP, the general health status of *C. callosus* improved, reducing cases of non-infectious lumbar alopecia. Therefore, EEP allows *C. callosus* to express natural reproductive behaviors and improves parental care.

## 1 Introduction

Animal models remain and will continue to be essential for scientific development for many years. Although, there is currently a crisis in pre-clinical biomedical research involving laboratory animals. This issue has been highlighted in many journals that publish irreproducible results. One estimate indicates that 28 billion dollars are wasted annually in the United States alone on unreliable research data due to lack of reproducibility (1). The causes of this reproducibility crisis can be numerous, such as methodological weaknesses (2), biological variability (3), and environmental factors (4). Nonetheless, due to the varying conditions under which animals are bred and maintained in experimental protocols at different institutions (5), the various improvements in the housing conditions of the animals through enrichment protocols not only enhance the reproducibility of studies using such animals but also improve their welfare, resulting in more reliable and accurate research outcomes (6).

Environmental enrichment seeks to improve the animals’ sensory and motor experiences by manipulating their living environment to increase social interaction, exploratory behavior, play, activity, and exercise levels. This type of enrichment involves exposing animals to environments rich in sensory stimulation, providing them with conditions that stimulate natural behavior and enhance comfort (7). Furthermore, environmental enrichment promotes well-being and enhances the quality of life of animals in confinement, used in experiments. It helps reduce physiological and behavioral changes caused by stress factors, which can negatively impact the reliability and reproducibility of research results.

**Figure 1:**
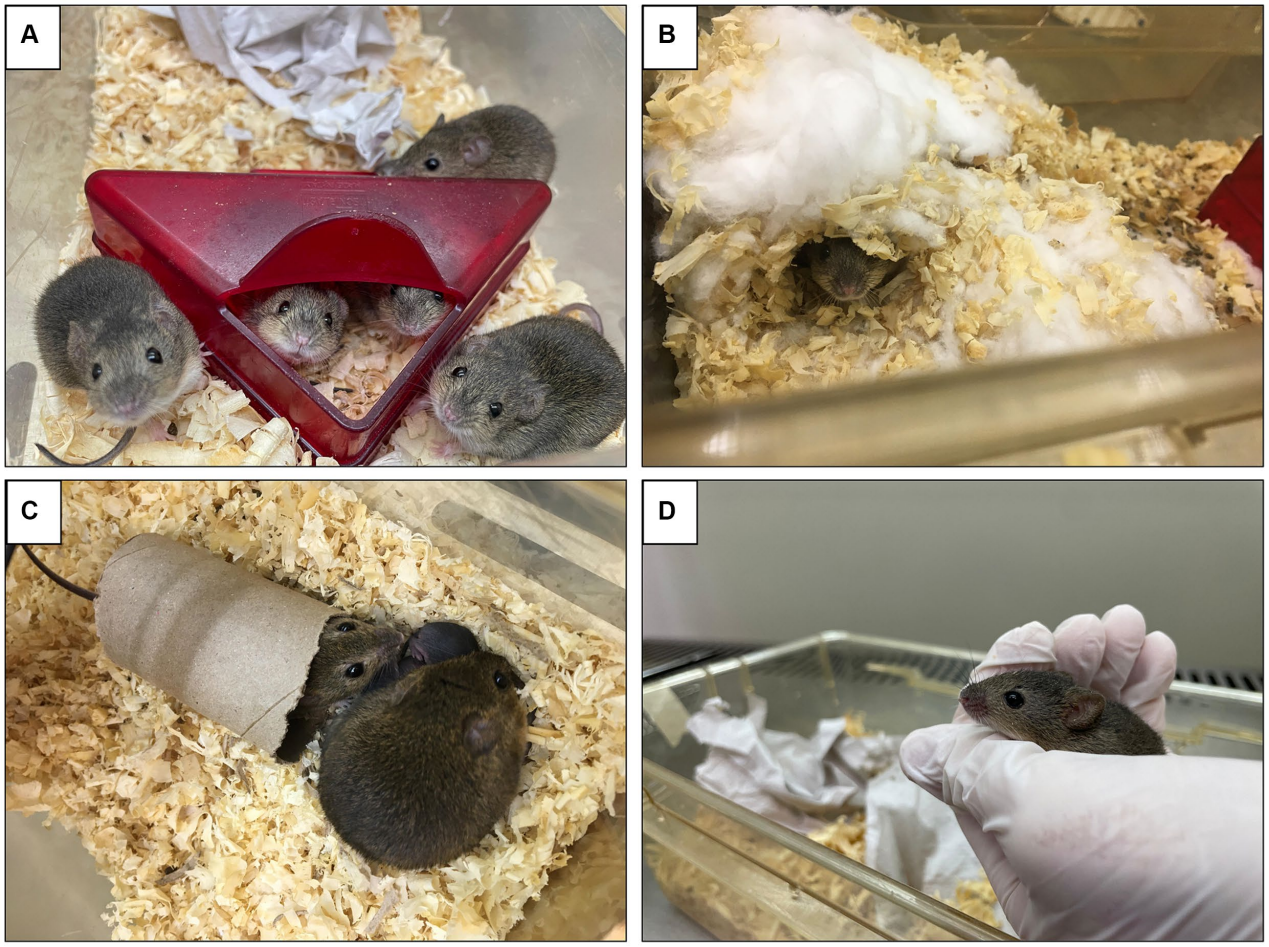
*Calomys callosus*, a member of the Cricetidae family, is a research animal model recently introduced into laboratories due to its ease of handling, longevity, and satisfactory prolificacy. The behavior of *C. callosus* in different environmental situations is depicted as follows: **(A)** Social interaction of the group in an igloo. **(B)** Burrow creation using hydrophobic cotton, sunflower seed packets, and wood shavings. **(C)** Hiding and navigating through a polypropylene roll, along with their nest of pups. **(D)** Handling of the animals, demonstrating their docile behavior. Photos are from the Central Bioterium of the Rodent Bioterium Complex (REBIR) at the Federal University of Uberlândia (UFU).

*Rattus rattus* (rats) and *Mus musculus* (mice) were introduced as laboratory animals at different times and for different purposes. An early report on the use of albino rats in research was made in 1856 by the renowned French physiologist Jean-Marie Philippeaux (8), and the first inbred rat strain was developed in 1906 at the Wistar Institute (Philadelphia, United States) (9). Additionally, around 1900, Abbie C. E. Lathrop supplied the first mice to several research laboratories, where they were bred and used by Lathrop and Castle (10). Conversely, Petter et al. (11) introduced *Calomys callosus* as a laboratory animal only in 1967 (Figure 1).

*Calomys callosus* is a rodent that is larger and heavier than a mouse, with more voluminous fur. Native to a region of South America, it belongs to the Cricetidae family (12). This terrestrial species inhabits dry and subhumid areas, such as the Chaco regions of Argentina, northeastern Bolivia, and Paraguay. In Brazil, it inhabits the central-western region with a tropical seasonal climate, including the vegetation of the “cerrado” *sensu stricto* (a type of savanna vegetation in central Brazil with high biodiversity and distinct dry and wet seasons), “cerradão” (a denser, forest-like cerrado vegetation with a more closed canopy), and deciduous forest (13, 14). Pairs of these rodents have been discovered in burrows about 1 ft long near water sources. They exhibit diurnal activity and feed on seeds and roots (13–15).

Compared to other rodents commonly used as experimental animals, *C. callosus* exhibits several distinct characteristics (15). For instance, it has less variable behavioral sequences and is less exploratory than *M. musculus* (16, 17); its offspring do not engage in play behavior (18); it shows good adaptation to food deprivation regimes (18); and males are less aggressive than albino *M. musculus* mice. However, they exhibit more aggression when faced with unfamiliar males than females in similar confrontations (19).

This South American rodent was introduced as a laboratory animal due to its specific characteristics, such as easy handling, longevity, and satisfactory prolificacy, making it a suitable model for efficiently replicating some parasitic and infectious diseases of public health interest, such as Toxoplasmosis, Leishmaniasis, Chagas, and Schistosomiasis (12). Since this species has proven to be an excellent experimental animal model, increasingly used in investigations of diseases relevant to public health, understanding the housing conditions for this species’ optimized well-being is fundamental for the reliability of research results.

The reproduction and sexual behavior of rodents are very important in animal facilities; however, they can be strongly influenced by environment, health, and nutrition (20). Since *C. callosus* was recently introduced as an animal model for research, and given our limited knowledge of this animal’s social behavior in its natural habitat, designing a suitable environment becomes increasingly challenging. Furthermore, one of the most significant factors allowing us to measure stress in such animals is the occurrence of reproductive parameter problems in the colony, which indicates a context of poor well-being (21). Therefore, creating an optimal captive breeding environment is essential to ensure the successful mating of these animals.

Unlike wild animals accustomed to life in a natural environment, various physiological and behavioral factors are altered when these animals are kept in a controlled environment such as a laboratory. The use of *C. callosus* in laboratory research is recent compared to other experimental rodent models, so we know little about its adaptation to captivity. An unsuitable environment for this species can cause chronic stress, leading to non-infectious alopecia (22, 23), which is a significant stress indicator commonly observed in *C. callosus* colonies. This stress negatively impacts reproduction and overall colony health, thus affecting experimental results (24). Other stress and anxiety behaviors include increased reactivity, aggressiveness, reduced grooming, and changes in nest structure (25).

Hence, an effective environmental enrichment program will ensure well-being, a good experimental design, and better research results. Accordingly, this study aimed to evaluate the impact of adopting a good Environmental Enrichment Program (EEP) for the *C. callosus* colony in the Central Animal Facility of the Rodents Animal Facilities Complex, Universidade Federal de Uberlândia (REBIR/UFU). It was predicted that the EPP could promote improved reproductive and postpartum zootechnical indices and reduced mortality.

## 2 Materials and methods

### 2.1 Animals and housing

*Calomys callosus* were bred and maintained under specific pathogen-free (SPF) conditions at the Central Animal Facility of the Rodents Animal Facilities Complex, Universidade Federal de Uberlândia (REBIR/UFU). They were housed in individually ventilated cages made of transparent polysulfone, model 1,285 L (Tecniplast©, Buguggiate, Italy; L × W × H, 365 mm × 207 mm × 130 mm; floor area 542 cm^2^), mounted on ventilated holding units. The room temperature was kept at 22–25°C (±2°C) and relative humidity at 45–65%. Lights were maintained on a 12:12 h cycle, with gradual transitions on at 06:00 and off at 18:00. The rodents were fed with dry food pellets, vacuum-packed, NUVILAB-CR1 (Quintia S.A©, Colombo, Paraná, Brazil), and provided with sterilized water through automatic drinking valves, both available *ad libitum*.

### 2.2 Breeding management

Nulliparous *C. callosus* females were monogamously paired with naïve males, aged 3 weeks. The breeding pairs were selected endogenously among siblings and were previously handled using the tail-lift method before study enrollment. These pairs were maintained together until the end of their breeding lifespan (6 months) to allow for continuous breeding. Pregnancy was visually checked daily based on physical appearance without disturbing the cage and during cage changes. Parturition was recorded from the first observation of neonatal pups. The total pup count was obtained within 1 day of birth by visual examination without opening the cage or disturbing the nest whenever possible. Pups were weaned at approximately 21 days. The removal of corpses was carried out immediately upon observation during daily inspections. The weaned pups were then distributed to institutional investigators at REBIR/UFU.

### 2.3 Experimental design

**Figure 2:**
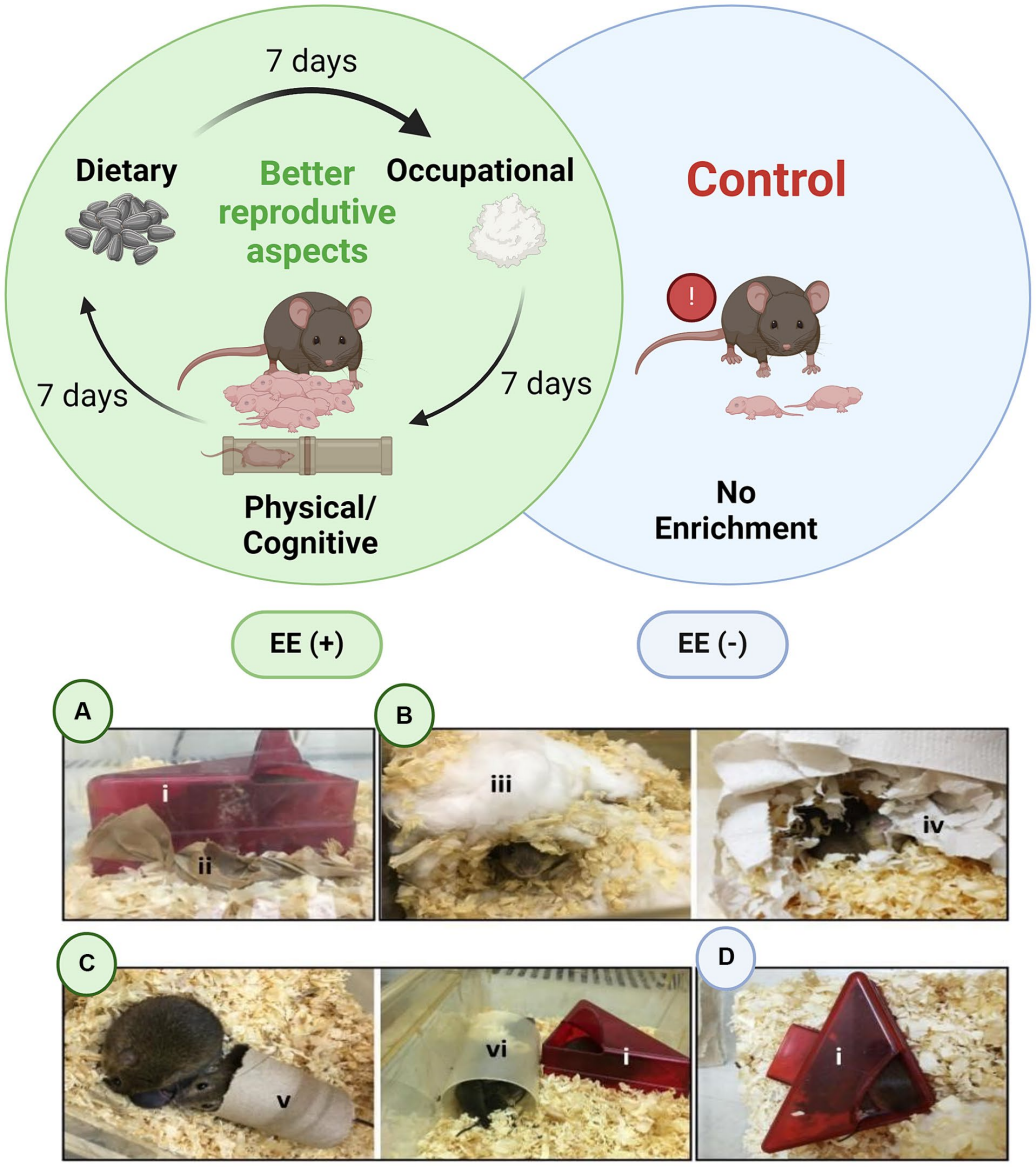
The Environmental Enrichment Program (EEP) was established at the Central Animal Facility of the Rodents Animal Facilities Complex, Universidade Federal de Uberlândia (UFU). The different enrichment groups are represented as follows: **(A)** Dietary enrichment, **(B)** Occupational enrichment, and **(C)** Physical/cognitive enrichment (**A–C** = EE+ group). **(D)** No enrichment/EE− group. The letters (i) refer to the enrichment materials: (i) Igloo, (ii) Sunflower seed packets, (iii) Hydrophobic cotton, (iv) Paper towel, (v) Paper roll, and (vi) Polypropylene roll. Diagram was created with BioRender (https://biorender.com/).

Two experimental groups were independently established: one with environmental enrichment EE+ and one without EE−, each consisting of at least 20 pairs of *C. callosus*. To better understand the standard breeding practices of *C. callosus* and determine if we were meeting the minimum welfare conditions, we experimentally outlined environmental enrichment. The control group EE− was maintained with only an igloo, food, and water provided *ad libitum*. The EE+ group was supplemented with various enrichment elements, as described below. The sequence of enrichments established in the EE+ group included dietary, occupational, and physical/cognitive enrichment. A new enrichment item was introduced every 11 days, and the previous one was removed. This weekly change of materials aimed to provide variation between the types of enrichment, considering the availability of materials (Figure 2).

Dietary enrichment was designed to be offered in small quantities and at low frequency (five sunflower seeds every 4 weeks for a box with at least two animals) to ensure it did not cause dietary changes, as the dry food pellets provided were sufficient. For dietary enrichment (Figure 2A), five sunflower seeds were wrapped in Kraft paper. Sunflower seed packets were added as a material for dietary enrichment, but they also served as physical/cognitive enrichment.

Figure 2B depicts how occupational enrichment was effectively accommodated using hydrophobic cotton, intended as nesting material. This provided comfort and insulation for the animals. Physical/cognitive enrichment (Figure 2C) consisted of tunnels, one made of paper and the other of polypropylene, where the animals could walk in and over, gnaw, take shelter, and express other behaviors. The igloo acted solely as a shelter and protective refuge for the animal. Once positioned and sustained within the enclosure, as depicted in Figure 2D, it became a permanent fixture in the environment, thus no longer considered environmental enrichment. Consequently, it transitioned from an enrichment element to a structural component within the enclosure, albeit indispensable for program implementation.

All EE materials were autoclaved before being offered to the animals. The differential diagnosis for non-infectious lumbar alopecia, compared to other skin diseases such as those caused by parasites, bacteria, fungi, and other nutritional and metabolic disorders, was investigated and ruled out (26). These parameters and others related to the animals’ health status were evaluated daily in the presence of a veterinarian and other qualified professionals (27). The behavior of *C. callosus* during nest building and their stay in these locations was evaluated observationally and collected casually or non-systematically.

Data from each pair of *C. callosus* regarding the first three births were used for each analysis. Postpartum zootechnical indices, such as the number of pups born alive, the number of pups weaned (evaluation period: 6 months or 24 weeks), and the number of deaths from birth to weaning, were measured daily (every 24 h) through manual inspection of the cages. Concurrently, data for the reproductive indices were collected. The effect of the EEP on the following parameters was evaluated and calculated as follows:

- Dam age when first litter was born = Σ(Dam age at first parturition_i)/*N*.
- Interval between parturitions of pups = Σ(Parturition Interval_i)/(*N* − 1).
- Percentage of breeding pairs who have not given birth to a pup in the previous 6 months = (Number of pairs without pups/Total number of pairs with pups) × 100%. Note: There is only one mean presented for each group.
- Number of pups born alive = Σ(Number of Live Births_i).
- Number of pups weaned [Evaluation period: 6 months (24 weeks) = Σ(Number of Weaned Pups_i)].
- Number of deaths from birth to weaning = Σ(Number of Deaths_i).

The number of experimental units evaluated per parameter is as follows: the dam age when the first litter was born had 86 experimental units; the interval between parturitions had 45 experimental units; the percentage of breeding pairs who have not given birth to a pup in the previous 6 months had 28 experimental units; the number of pups born alive had 139 experimental units; the number of pups weaned had 139 experimental units; and the number of deaths from birth to weaning had 139 experimental units. More details of the equations are presented in Supplementary material 1.

### 2.4 Statistical analysis

Statistical analyses were performed using GraphPad Prism 10 software (GraphPad Software Inc., United States). We assessed the data distribution using normality and lognormality tests. For normally distributed data, we used the T-test for independent groups or the paired T-test for related samples. We used the Mann–Whitney test for independent groups or the Wilcoxon test for non-normal data for paired samples. Levene’s test was used to check variance homogeneity before the T-test. More detailed information is in Supplementary material 1.

## 3 Results

**Figure 3:**
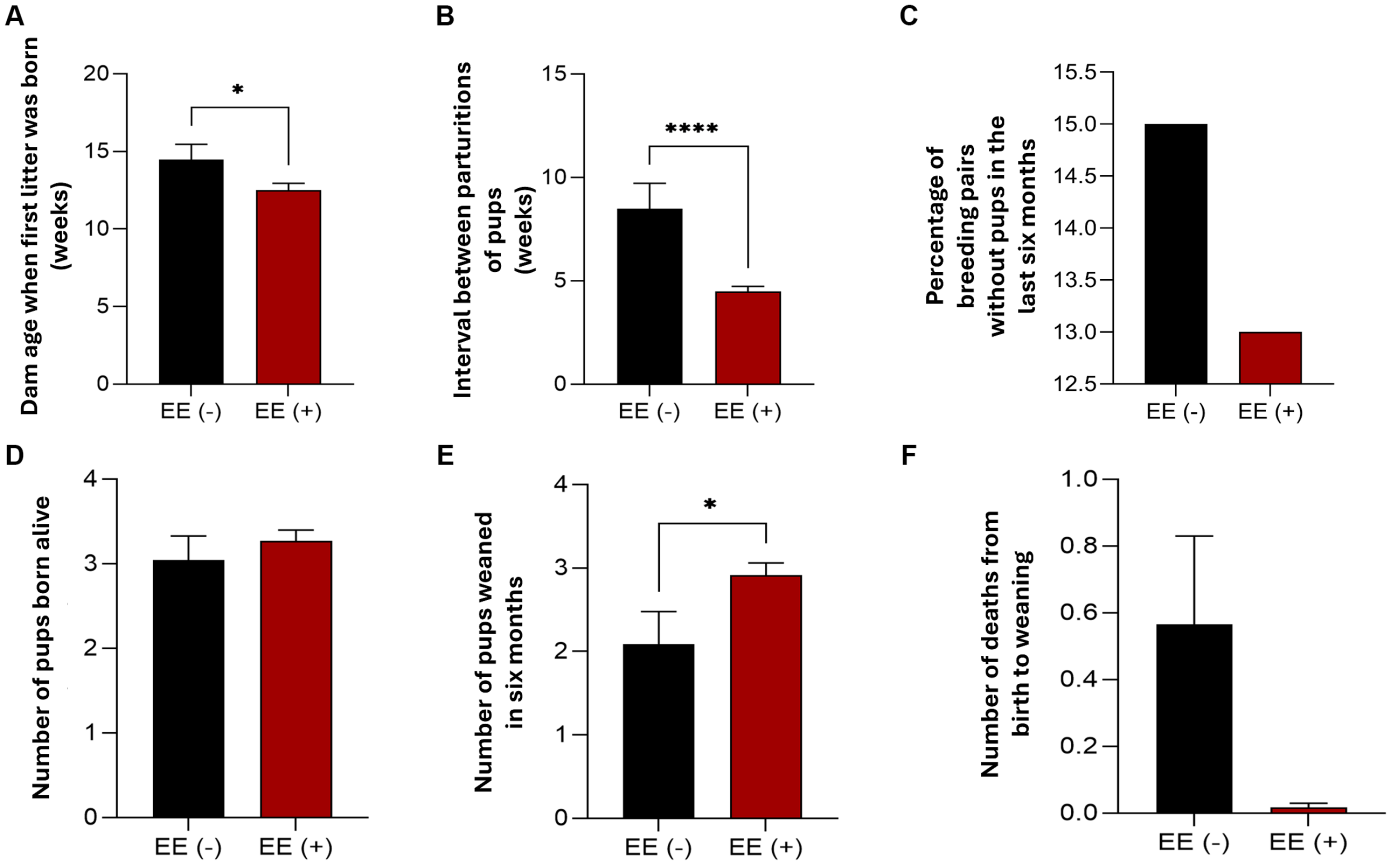
The effects of the Environmental Enrichment Program (EEP) on reproductive and postpartum zootechnical indices in *Calomys callosus* were evaluated with and without environmental enrichment (EE+ and EE−, respectively). The parameters assessed included: **(A)** age of the dam at first litter birth, **(B)** interval between pup parturitions, **(C)** percentage of breeding pairs not giving birth to a pup in the previous 6 months, **(D)** number of pups born alive, **(E)** number of pups weaned (evaluation period: 6 months, 24 weeks), and **(F)** number of deaths from birth to weaning. Results are expressed as the mean ± standard error of the mean (SEM). Data were analyzed using the unpaired *t*-test, with differences considered significant at *p* values of <0.0001 (****) and <0.05 (*).

Our data demonstrate that environmental enrichment EE+ improved reproductive precocity in *C. callosus* females, as evidenced by a reduction in the age at first parturition. This was shown by the “Dam age when first litter was born” for the EE+ group (Figure 3A) compared to females without environmental enrichment EE− [two-tailed; *t*(72) = 2.013, *p* = 0.0479]. Females under EE+ management also exhibited a notable decrease in the “Interval between parturitions of pups” compared to EE− (Figure 3B) [two-tailed; *t*(43) = 4.811, *p* < 0.0001]. Fewer pairs failed to produce offspring over the 24 weeks in the presence of EE+ (Figure 3C). In other words, the observed trend indicated that pairs exposed to EE+ generated more offspring over the evaluated period.

The data demonstrate that EE+ did not change the number of animals born alive [two-tailed; *t*(137) = 0.7013, *p* = 0.4843] (Figure 3D). However, EE+ increased the number of pups weaned in 6 months (Stats, Figure 3E) and reduced the average number of deaths from birth to weaning compared to the absence of environmental enrichment (Stats, Figure 3F).

**Figure 4:**
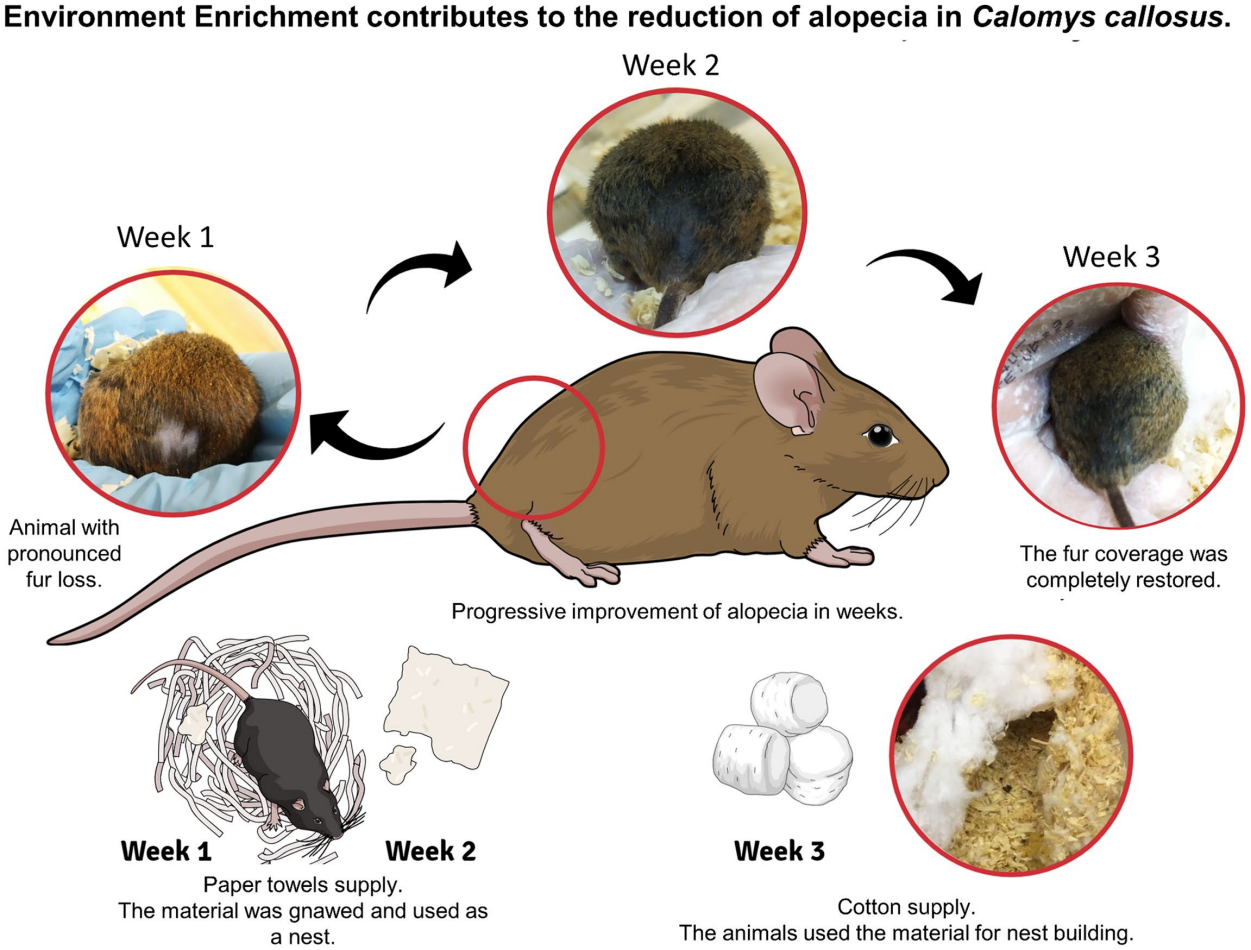
Demonstration of the changes in “fur coverage” in *Calomys callosus*, subjected to continuous Environmental Enrichment Programs (EEP) and diagnosed with non-infectious lumbar alopecia. During the weeks of the study, materials used in environmental enrichment were changed, and the animals used them to build nests. Diagram created in the Mind the Graph platform (www.mindthegraph.com).

Furthermore, after the inclusion of the EEP, the general health status of *C. callosus* improved. Specifically, in two cases of non-infectious lumbar alopecia, the animals spontaneously recovered completely from symptoms after 3 weeks of enrichment, with no new reports in the populations studied (Figure 4). This reduced the number of new cases to zero. It was casually observed that *C. callosus* has a pronounced tendency to hide, even in EE+. Notably, no cases of cannibalism were observed.

## 4 Discussion

In this study, the reproductive data of *C. callosus*, with EE+ and without EE− an environmental enrichment program, were analyzed, and t Diagram created in the Mind the Graph platform (www.mindthegraph.com).he differences between the two groups were examined. The implementation of the program in the EE+ group resulted in an earlier age at first parturition compared to the EE− group, a significant reduction in the interval between parturitions, and an increase in the number of weaned pups per litter. This demonstrates that the EE+ positively affected the reproduction of *C. callosus*. One of the main factors that likely contributed to this improvement was the provision of nesting materials, which allowed the pairs of *C. callosus* to enhance their maternal care by building nests to shelter their pups, keeping them warm and comfortable (28).

Although we did not record the nest scores for our animals, we observed that *C. callosus* managed to build quite elaborate nests using paper towels and cotton. Many of these nests were completely closed, with only a small, rounded opening for external access, characteristic of the highest score described by Gaskill et al. (29). In addition to providing warmth, these closed nests allow the animals to hide and create a sense of security. *Calomys callosus* has a very marked characteristic of hiding, and allowing resources to express this behavior certainly contributes to their well-being. Moreira et al. (30) found that providing nesting materials for Swiss Webster mouse pairs, such as polypropylene caps and cotton, improves maternal and paternal behaviors, such as pup licking and resting in contact with pups, and reduces non-contact resting behaviors. This indicates that offering a safer environment for animals, where they can take refuge if they feel threatened, promotes better parental care, higher survival rates, and reduced perinatal death.

Studies based on observations indicate that *C. callosus* may feed on seeds and roots (31, 32). The sunflower seed packets, in turn, offered a form of exploration with a reward, providing dietary and cognitive enrichment. Besides the behavioral benefits, they provided additional fat nutritional support, which may benefit pregnant and lactating females. However, although the nutritional needs of *C. callosus* are not well described in the literature, some studies with mice suggest that females in these stages require higher proportions of fats and proteins (33). This nutritional support could even reduce neonaticide rates (34, 35). Of similar importance, cannibalism is rarely observed in *C. callosus* (36), exhibiting behaviors similar to those observed in laboratory mice (37), as noted in this study.

Moreover, decreased intervals between parturitions and an earlier age at first litter observed in this study are likely linked to enrichment’s greater comfort and environmental improvements. As such, more dynamic, interactive, and natural-like environments are likely to decrease corticosterone levels, keeping them closer to baseline, thus reducing interference with sex hormones and consequently enhancing reproduction (38). Furthermore, the interval between births in females of the *C. callosus* EE+ group was reduced compared to those in the EE− group. This result is likely due to increased animal comfort and reduced stress with the EE+. Accordingly, an inadequate macroenvironment can affect the estrous cycle of female rats, as various factors can interfere with gestation time and ovulatory rate, which may be related to the present study’s findings (39).

Besides that, we observed a reduction in mortality, which can be associated with improvements in reproductive parameters and reproductive indices (40, 41). Hence, this study supports that the availability of shelter materials contributes to a safer and more comfortable environment, allowing *C. callosus* to express their natural behaviors better and improve parental care. Consequently, this improvement encompasses aspects such as nursing, pup warmth, and the reduction of perinatal death, among other factors that ultimately influence survival rates. Another noteworthy result from this study was that including EE+ improved the overall clinical health status of *C. callosus*, eliminating cases of non-infectious lumbar alopecia in the colonies. This corroborates Bechard et al. (42), who observed lower prevalence and severity of alopecia in enriched cages compared to non-enriched ones in a study with C57BL/6 J mice.

Likewise, other factors such as cage size and stocking density (cage area per mouse) may play a crucial role in triggering alopecia in these animals (35, 38, 39, 43, 44). However, our study did not control for animal stocking density between treatments, although at least one previous study found that animal density did not affect coat cover (44). On the other hand, the size of cages can be a factor that improves the reduction of alopecia, indicating that increasing cage size may contribute positively to the disappearance of these symptoms (35). In future studies, we will consider evaluating these environmental factors.

## 5 Conclusion

In this study, we have demonstrated significant enhancements in the welfare of *C. callosus* by implementing our Environmental Enrichment Program (EEP) within rodent facilities. Our findings indicate that the EE+ yields notable improvements in reproductive outcomes, including reductions in dam age at first parturition, intervals between parturitions, and the percentage of breeding pairs that have not given birth to a pup in the previous 6 months. While the EE+ does not significantly impact the overall number of pups born alive, it positively influences the number of pups weaned per birth. It concurrently reduces mortality rates from birth to weaning. Given the complexities of these dynamic interactions, further investigations into novel forms of environmental enrichment tailored to *C. callosus* are warranted. These efforts promise continued advancements in optimizing this valuable research species’ welfare and breeding success.

## Data Availability

The original contributions presented in the study are included in the article/Supplementary material, further investigations can be directed to the corresponding author.
