# Supplementary material for: A simple and low-cost environmental enrichment program improves the welfare of Calomys callosus, a species that adapts to animal facilities
Source: Front Vet Sci. 2024 Sep 13;11:1436907. doi: 10.3389/fvets.2024.1436907 (PMC11428199; doi:10.3389/fvets.2024.1436907)
Supplement: Supplementary file 1 [file Table_1.docx]

Supplementary Material

**A simple and low-cost environmental enrichment program improves the welfare of *Calomys* *callosus*, a species that adapts to animal facilities.**

Sandra Gabriela Klein^1^, Tamires Soares de Assis1, Gabriel Silva Pereira^2^, Loyane Bertagnolli Coutinho^2^, Renan Faria Guerra^2^, Matheus Morais Neves^1^, Flávia Batista Ferreira^1^, Isabela Lemos de Lima^1^, Richard Costa Polveiro^1^, Eloisa Amália Vieira Ferro^3^, Murilo Vieira da Silva^1,2^*

^1^Biotechnology in Experimental Models Laboratory - LABME, Universidade Federal de Uberlândia, Uberlândia, Brazil, ^2^Rodents Animal Facilities Complex, Universidade Federal de Uberlândia, Uberlandia, Brazil, ^3^Institute of Biomedical Sciences, Universidade Federal de Uberlândia, Uberlandia, Brazil.

*** Correspondence:**Murilo Vieira da Silva
[murilo.vieira@ufu.br](mailto:murilo.vieira@ufu.br)

**Supplementary Data 1**

**Age at First Parturition (Independent variable) - (Two Experimental groups / 86 Experimental units):**

- Formula: Dam age when first litter was born = Σ (Dam age at First Parturition_i) / N

Σ (Sigma): Represents the sum of all individual values.

Dam age at First Parturition_i: Age of the i-th dam when she gave birth for the first time.

N: Total number of dams in the study who gave birth at least once.

**Interval Between Parturitions (Independent variable) - (Two Experimental groups / 45 Experimental units): Average: Time between successive parturitions for each dam.**

- Formula: Interval between parturitions of pups = Σ (Parturition Interval_i) / (N -1)

Parturition Interval_i: Time interval between the i-th and (i+1)-th parturition for a particular dam.

Dams with only one parturition are excluded (hence N−1).

**Percentage of Pairs of Reproductive Age Who Had Not Produced Offspring for Six Months (Dependent variable) - (Two Experimental groups / 28 Experimental units): The proportion of breeding pairs in the study that haven't had offspring within the past six months.**

- Formula: Percentage of breeding pairs who have not given birth to a pup in the previous six months = (Number of pairs without pups / Total number of pairs with pups) x 100%

Number of Pairs Without Pups: Number of pairs that haven't had any pups born alive in the past six months.

**Number of Animals Born Alive (Independent variable) - (Two Experimental groups / 139 Experimental units): The total number of pups born alive during the study period.**

- Formula: Number of pups born alive = Σ (Number of Live Parturition Interval_i)

Number of Live Parturition Interval_i: Number of live births for the i-th dam or breeding pair.

Evaluation period: Six months (24 weeks).

**Number of Weaned Pups (Dependent variable) - (Two Experimental groups / 139 Experimental units): Total number of pups that successfully survived to weaning age.**

- Formula: Number of pups weaned = Σ (Number of Weaned Pups_i)

Number of Weaned Pups_i: Number of pups for the i-th dam or breeding pair that reached weaning age.

**Number of Deaths from Birth to Weaning (Dependent variable) - (Two Experimental groups / 139 Experimental units)**: The total number of pups that died between birth and weaning.

- Formula: Number of deaths = Σ (Number of Deaths_i)

Number of Deaths_i: Number of pups that died between birth and weaning for the i-th dam or breeding pair.

**The normal distribution of group data was analyzed using the D'Agostino & Pearson and Kolmogorov-Smirnov tests (statistical analysis with 95% confidence).**

- Dam age when first litter was born**:** Lognormal distribution.
- Interval between parturitions of pups: Normal distribution by Kolmogorov-Smirnov.
- Percentage of breeding pairs who have not given birth to a pup in the previous six months: A normality test is not required, as it is a comparison of two reference averages.
- Number of pups born alive: Normal distribution by D'Agostino.
- Number of pups weaned: Normal distribution by D'Agostino.
- Number of deaths from birth to weaning: Non-normal distribution, but tests indicate high data reliability.
